# Positivity of HIV, hepatitis B and hepatitis C in patients enrolled in a confidential self-exclusion system of blood donation: a cross-sectional analytical study

**DOI:** 10.1590/S1516-31802010000600002

**Published:** 2010-12-02

**Authors:** Leila Kasraian, Alireza Tavasoli

**Affiliations:** I MD. Manager of Education and Research Department, Shiraz Blood Transfusion Organization, Fars, Iran.; II MD. Pathologist and President of Shiraz Blood Transfusion Organization, Fars, Iran.

**Keywords:** Blood donors, Blood-borne pathogens, Safety, Hepatitis, HIV, Donadores de sangre, Patógenos transmitidos por la sangre, Seguridad, Hepatitis, VIH

## Abstract

**CONTEXT AND OBJECTIVE:**

Selection of healthy blood donors is essential to ensure blood safety. A confidential self-exclusion (CSE) system was designed so that high-risk donors could confidentially exclude their blood from use in transfusions. This study aimed to compare the demographic characteristics and the results from human immunodeficiency virus (HIV), hepatitis B surface (HBS) and hepatitis C virus (HCV) screening tests on donors who opted to get into and out of CSE.

**DESIGN AND SETTING:**

Analytical cross-sectional study on all volunteer donors at Shiraz Blood Transfusion Organization from March 21, 2006, to March 21, 2008.

**METHODS:**

The results from the abovementioned tests were compared between donors who opted into and out of CSE.

**RESULTS:**

100,148 donors in 2006 and 104,271 in 2007 gave blood. Among these donors, respectively, 829 (0.82%) and 592 (0.57%) opted for the CSE. The prevalence of HIV antibodies, HBS antigens and HCV antibodies in CSE donors was significantly higher than in donors who did not choose CSE (P < 0.05). The prevalence of at least one of these three infections among CSE donors was 3.12% in 2006 and 3.04% in 2007, and was significantly higher than the prevalence among non-CSE donors (0.58% and 0.57%, respectively).

**CONCLUSION:**

Because of the higher prevalence of HBS, HCV and HIV positivity in blood donors who chose the CSE option, offering CSE to blood donors could be a potentially useful method for improving blood safety, since it could increase the detection of infected blood during the window period.

## RESUMEN

**CONTEXTO Y OBJETIVO:**

La identificación y selección de donantes de sangre sanos es el primer paso para asegurar la seguridad de la sangre. Este estudio tiene como objetivo comparar las características demográficas y los resultados de los virus de la inmunodeficiencia humana (VIH), la superficie de la hepatitis B (HBS) y viru de la hepatitis C (VHC) las pruebas de cribado en los donantes que optaron por entrar y salir de la libre y confidencial de exclusión.

**DISEÑO Y EMPLAZAMIENTO:**

Estudio transversal de todos los donantes voluntarios que acudieron a la Shiraz Blood Transfusion Organization entre el 21 de Marzo del 2006 y el 21 de Marzo del 2008.

**MÉTODOS:**

Los resultados de las pruebas antes mencionadas fueron comparados entre los donantes que optaron por entrar y salir de CSE.

**RESULTADOS:**

100.148 donantes en 2006 y 104.271 en 2007 donaron sangre. Entre estos donantes, respectivamente, 829 (0,82%) y 592 (0,57%) optaron por el CSE. La prevalencia de anticuerpos contra el VIH, los antígenos HBS y anticuerpos contra el VHC en donantes de CSE fue significativamente mayor que en los donantes que no eligió CSE (P < 0,05). La prevalencia de al menos una de estas tres infecciones entre los donantes CSE fue 3,12% en 2006 y 3,04% en 2007, y fue significativamente mayor que la prevalencia entre los donantes no CSE (0,58% y 0,57%, respectivamente).

**CONCLUSIONES:**

Debido a la mayor prevalencia de BA, el VHC y la infección con VIH en donantes de sangre que eligieron la opción del CSE, ofreciendo CSE para los donantes de sangre podría ser un método potencialmente útil para mejorar la seguridad de la sangre, ya que podría aumentar la detección de sangre infectada durante el período de ventana.

## INTRODUCTION

Blood donation safety is a major concern of blood transfusion organizations.^1^ Identification and selection of healthy blood donors is the first step towards ensuring blood safety.^2^ Some volunteers do not divulge information about their high-risk behavior in their interview with the physician/nurse at the donation center. However, these individuals may already be aware that they are not suitable for donation, but they still wish to donate their blood. Their motivation for wanting to donate may be based on the belief that donating will have positive effects on their health status, the desire to receive a health check-up, or pressure from friends or family to know their HIV status. The main underlying reason for this problem is that testing for HIV is free of charge and fast at blood centers and people wish to avoid the social stigmatization of specifically being tested for HIV.^3,4^

Confidential self-exclusion (CSE) systems have been designed for this type of blood donor, so that high-risk donors can confidentially exclude their blood from use for transfusions.^1^ The first CSE system was designed in 1984 in the United States as an option for improving blood safety,^1^ and similar systems have since been used in many countries.^1^ At the Fars Province Blood Transfusion Organization in Shiraz (southern Iran), a CSE system was launched in 2006. The system has been used as an extra screening test to detect blood-borne diseases in donors who come to the donation center during their window period.^1,5-7^

However, earlier studies suggested that the sensitivity and positive predictive value of CSE may be low.^8^ In 1992, the Food and Drug Administration (FDA) recommended that CSE should not be used.^9^

In contrast, the Australian Research Center has continued to use a CSE system because it may enhance blood safety.^10^ Over recent decades, blood safety has been improved noticeably through the recruitment of safe blood donors and the use of sensitive screening tests and shorter window periods.^5,6^

## OBJECTIVE

This study was designed to compare the demographic characteristics and the results from human immunodeficiency virus (HIV), hepatitis B surface (HBS) and hepatitis C virus (HCV) screening tests in donors who opted into and out of confidential self-exclusion.

## METHODS

The participants in this cross-sectional study were all volunteer blood donors who donated blood at our center between March 21, 2006 and March 21, 2008. The donors were interviewed by physicians before donation and information about any high-risk behavior was obtained. Shiraz Blood Transfusion Organization uses a standard form for CSE, and all physicians have been instructed to inform blood donors about the CSE option. After donation, they gave the form to the donors so that they could opt into or out of CSE. The donors filled out the form confidentially and put it in the box designed for this purpose.

The blood units were examined for HIV antigen-antibodies (Bio-Rad, Coquette, France), hepatitis C virus (HCV) antibodies (Ortho, New Jersey, USA) and hepatitis B surface (HBS) antigens (Behring, Marburg, Germany) by means of the enzyme-linked immunosorbent assay (ELISA). All the positive results were confirmed by means of western blot for HIV (Inonogenetic, Ghent, Belgium), recombinant immunoblot assay (RIBA) for HCV (Inonogenetic, Ghent, Belgium) and the neutralization test (Behring, Marburg, Germany) for hepatitis B virus (HBV).

This survey was performed on all donors. There were 583 instances of missing data in 2006 and 415 instances in 2007, which were due to missing blood samples. The institution's ethics committee gave approval for the survey and assurances were provided that the information would be kept confidential.

The demographic characteristics and the results from the screening tests on the blood given by the donors who opted into and out of CSE were compared to determine the prevalence of HIV, HBS and HCV positivity in the two groups. We calculated the sensitivity, specificity, positive predictive value (PPV) and negative predictive value (NPV) of CSE. Sensitivity was defined as the proportion of CSE donors out of all donors who were identified as being positive or seroconverted. Specificity was calculated as the proportion of the donors who opted out of CSE and who were identified as being negative. The PPV was the proportion of positive donors who opted into CSE. The chi-square test and Fisher's exact test were used to compare the values between the groups. The MedCalc for Windows 7.0 test was used to compare the values between the groups.

## RESULTS

During the study period, 100,148 donors in 2006 and 104,271 in 2007 gave blood at our center. Among these, 829 donors (0.82%) and 592 (0.57%), respectively, opted into CSE. The prevalence of HIV antibodies, HBS antigens and HCV antibodies in the CSE donors’ blood was significantly higher than in the blood of the donors who did not choose self-exclusion (p < 0.05) (Table 1).

**Table 1.** Prevalence of hepatitis B surface (HBS) antigen, hepatitis C virus (HCV) antibody and human immunodeficiency virus (HIV) antibody in blood donors who opted into and out of confidential self-exclusion

Blood test	Year	Confidential self-exclusion	Number of donors	Number of positive cases	Disease prevalence	P Value
HIV antibody	2006	No	99319	11	0.011%	< 0.001
		Yes	829	2	0.24%	
	2007	No	103679	10	0.009	< 0.001
		Yes	592	2	0.34%	
HBS antigen	2006	No	99319	382	0.38%	< 0.001
		Yes	829	16	1.9%	
	2007	No	103679	381	0.36%	< 0.001
		Yes	592	8	1.35%	
HCV antibody	2006	No	99319	185	0.18%	< 0.001
		Yes	829	8	0.96%	
	2007	No	103679	206	0.19%	< 0.001
		Yes	592	8	1.35%	

The prevalence of findings of at least one infection out of the three blood-borne diseases in CSE donors was 3.14% in 2006 and 3.04% in 2007, and these rates were significantly higher than in non-CSE donors (0.58% and 0.57%, respectively; p < 0.05). The odds of the presence of blood-transmitted disease in the CSE group in 2006 and 2007 were 5.35 (CI 3.59-7.98; P < 0.01) and 5.28(CI 3.28-8.49; P < 0.01), compared with the non-CSE group.

There were no differences in the prevalences of HIV antibodies, HCV antibodies and HBS antigens in donors who chose CSE, either between men and women (Table 2) or between first-time and previous donors (Table 3) (P > 0.05). The sensitivity of CSE for HIV antibodies, HBS antigens and HCV antibodies was 16.0%, 3.04% and 3.93%, respectively, and the specificity was 99.0%, 99.3% and 99.3%, respectively. The PPV of CSE for HIV antibodies, HBS antigens and HCV antibodies was 0.28%, 1.68% and 1.12%, and the NPV was 99.98%, 99.6% and 99.8%, respectively.

**Table 2.** Prevalence of hepatitis B surface (HBS) antigen, hepatitis C virus (HCV) antibody and human immunodeficiency virus (HIV) antibody in male and female blood donors who opted into confidential self-exclusion

Year	2006	Donation status	+	-	odds ratio	confidence interval
				HCV			
		male	7	775	2.4	0.29-19.9
		female	1	46		
			HBS			
		male	16	766	0.96	0.125-7.46
		female	0	47		
			HIV			
		male	2	780	0.12	0.01-1.35
		female	0	47		
	2007		HCV			
		male	7	556	0.3	0.04-2.9
		female	1	28		
			HBS			
		male	7	556	0.3	0.04-2.9
		female	1	28		
			HIV			
		male	2	561	0.103	0.009-1.17
		female	0	29		

Reference level is female donors.

**Table 3.** Prevalence of hepatitis B surface (HBS) antigen, hepatitis C virus (HCV) antibody and human immunodeficiency virus (HIV) antibody in blood donors who opted into confidential self-exclusion (CSE), based on blood donation status (first-time versus repeated blood donors)

Year	2006	Donation status	+	-	odds ratio	confidence interval
				HCV			
		first-time blood donors	8	534	4.29	0.5-34.54
		repeated blood donors	0	287		
			HBS			
		first-time blood donors	14	526	8.7	1.15-66.16
		repeated blood donors	2	287		
			HIV			
		first-time blood donors	2	540	1.06	0.1-11.77
		repeated blood donors	0	287		
	2007		HCV			
		first-time blood donors	8	316	6.7	0.8-54.59
		repeated blood donors	0	268		
			HBS			
		first-time blood donors	8	316	6.7	0.8-54.59
		repeated blood donors	0	268		
			HIV			
		first-time blood donors	2	322	1.66	0.15-18.45
		repeated blood donors	0	268		

Reference level is repeated donors.

## DISCUSSION

In this survey, the prevalence of HBS, HCV and HIV in CSE blood donors was significantly higher than that in non-CSE donors. Some studies have reported the same finding as that of the study by Pindyck et al.^1^ Another study by Petersen et al. showed that the prevalence of HIV antibodies in those who confidentially excluded their blood was 21 times as high as in the donors who did not.^7^ Another study showed that CSE donors had a higher prevalence of HBS, HCV and HIV, and the use of the CSE option was estimated to prevent the collection of 0.2 to 1.3 window-period units annually.^10^ In a study by Koerner et al., the prevalence of HBS antibodies in male blood donors who excluded their blood was higher.^11^ Chiewsilp showed that the prevalence of HBV, HCV and HIV was greater in CSE donors.^12^

In this study, the sensitivity and positive predictive value of CSE were low, and similar to previous studies.^8-10^ Moreover, 0.7% of our blood donors chose to exclude their blood from further use. In a study by Pastucha et al., 1.3% of the blood donors excluded their blood, a figure that was around twice as much as the proportion in our study.^6^ This may reflect differences between earlier studies and ours, regarding the amount of information on the importance of voluntary self-exclusion that was provided to donors. In spite of the higher prevalence of blood borne diseases in CSE donors, this method may have had a minimal effect on blood safety in this study because of the small number of donors who chose the CSE option in our setting. This was similar to the study by Petersen et al.^7^

One of the benefits of a CSE system is that it allows blood donors to decide whether their blood will be used for transfusion; this choice may increase their sense of responsibility and thus improve blood safety. The other benefit of this method is its low cost. The CSE option may be a useful method in our setting (Iran), where some donors may donate for the positive effect of blood donation on their health or may donate to obtain an HIV test, given that testing in this center is free and that, due to social or cultural pressure, they have a tendency to conceal their high-risk behavior.^3^ On the other hand, one negative aspect of this system is that some safe blood units may be wasted if donors misunderstand the purpose of CSE and choose this option when they are not in fact at high risk of blood-borne diseases.

One of the limitations of this survey is that we do not have any information about donors who chose the CSE option and whether donors with negative screening results later become positive or not. We therefore suggest that a longitudinal study should be conducted in order to investigate seroconversion among CSE donors.

## CONCLUSIONS

Even though we were not able to detect an impact with our small sample, due to the higher prevalence of HBS, HCV and HIV positivity in blood donors who chose the CSE option, offering CSE to blood donors could be a potentially useful method for improving blood safety, since this could increase the detection of infected blood during the window period.
